# A comprehensive morphological database of hognose *Porthidium* pitvipers (Viperidae: Crotalinae)

**DOI:** 10.1093/database/baaf085

**Published:** 2026-01-19

**Authors:** Carlos Patron-Rivero, Carlos Yañez-Arenas, Sara Ruane, Xavier Chiappa-Carrara, Octavio R Rojas-Soto

**Affiliations:** Laboratorio de Ecología Geográfica, Unidad de Conservación de la Biodiversidad, UMDI-Sisal, Facultad de Ciencias, Universidad Nacional Autónoma de México, Sierra Papacal, Yucatán 97302, México; Life Sciences Section, Negaunee Integrative Research Center, Field Museum, Chicago, IL 60605, United States; Laboratorio de Ecología Geográfica, Unidad de Conservación de la Biodiversidad, UMDI-Sisal, Facultad de Ciencias, Universidad Nacional Autónoma de México, Sierra Papacal, Yucatán 97302, México; Life Sciences Section, Negaunee Integrative Research Center, Field Museum, Chicago, IL 60605, United States; Laboratorio de Ecología Geográfica, Unidad de Conservación de la Biodiversidad, UMDI-Sisal, Facultad de Ciencias, Universidad Nacional Autónoma de México, Sierra Papacal, Yucatán 97302, México; Departamento de Sistemas y Procesos Naturales, Escuela Nacional de Estudios Superiores Unidad Mérida, Universidad Nacional Autónoma de México, Yucatán 97357, México; Laboratorio de Bioclimatología, Red de Biología Evolutiva, Instituto de Ecología A.C. Carretera Antigua a Coatepec 351, El Haya 91073 Xalapa, Veracruz, México

## Abstract

Generating and sharing primary biological data is essential to support reproducible research, stimulate new hypotheses, and advance our understanding of biodiversity. Here, we present a comprehensive database of morphological traits for snakes of the genus *Porthidium* (Viperidae: Crotalinae). This database includes linear measurements, pholidosis (scale counts), and head shape data from preserved specimens across five different herpetological collections. These data comprise 13 morphological traits, 8 scale counts, and 55 landmarks collected from 484 individuals across 9 species. The specimens represent both juvenile and adult stages. All data were collected using standardized protocols to ensure comparability across individuals and species. The dataset is a valuable resource for studies in systematics, morphological evolution, ecological adaptation, and ontogeny, as well as facilitating reproducibility and reuse in the fields of evolutionary biology, herpetology, and comparative morphology.

## Introduction

Understanding the processes that shape phenotypic diversity is central to ecology and evolutionary biology [1]. In snakes, morphological traits—such as body proportions, scale counts, and head shape—are not only essential for species identification, but also reflect functional adaptations that influence thermoregulation, locomotion, feeding, and defensive behaviours [2]. Documenting morphological variation therefore provides critical insights into evolutionary constraints, ecological strategies, and the adaptive potential of species across heterogeneous environments.

Despite their ecological and evolutionary relevance, morphological data for many species remain scarce, scattered, or confined to qualitative taxonomic descriptions. While foundational syntheses provide a valuable baseline of information for many species [3], detailed, standardized datasets that span multiple developmental stages, species, and geographic ranges are rare [4, 5], limiting reproducibility and the ability to investigate macroevolutionary patterns, assess morphological disparity, or link phenotypic variation to environmental gradients [6]. These gaps range from knowing the basic geographic distribution of species and their evolutionary relationships, to understanding their environmental tolerances. Most importantly, this data scarcity limits our ability to link species-specific morphological and functional information to broader ecological and evolutionary patterns (see [7, 8]).

The hognose pitvipers of the genus *Porthidium* (Viperidae: Crotalinae) comprise nine recognized species distributed from Mexico to northern South America; this genus exhibits remarkable ecological and morphological diversity [9]. However, despite their distinctive morphology and medical relevance as venomous snakes, the genus presents persistent taxonomic uncertainties and a fragmented morphological knowledge base. Many existing species descriptions within *Porthidium* are outdated and in need of modern taxonomic revision. For instance, the *P. lansbergii*, first described in 1841 [10], has not received a comprehensive update since the elevation of *P. arcosae* to species status in 1993 [11]. Other species also lack recent redescription, such as *P. nasutum*, despite known controversies involving misidentifications with *Bothrops asper* and a complex sympatric intergrade zone with *P. yucatanicum* [12]. Similarly, recent evidence suggesting a possible allopatric speciation process in *P. dunni*, involving populations split by the Isthmus of Tehuantepec [9], further underscores the need for updated morphological assessments, for which a robust morphological dataset is crucial for integrative taxonomic resolution. The current scarcity of standardized, species-level morphological data for *Porthidium* directly impedes research into its evolutionary history, ecomorphology, and niche partitioning among sympatric species.

Hence, high-quality, openly accessible morphological datasets are powerful resources for both fundamental and complex research. They enable reproducible analyses, facilitate integration into phylogenetic, functional, and community ecology frameworks, and provide proxies for ecological functions where behavioural or physiological data are lacking—particularly for elusive or understudied species. By generating and sharing such datasets, researchers can enhance taxonomic resolution, strengthen comparative studies, and promote collaboration across disciplines. Here, we present a comprehensive, publicly available morphological dataset for *Porthidium* that includes all currently described species, which we hope enhances the scientific output and understanding for these snakes across the global research community.

## Materials and methods

### Taxon sampling

We examined 484 *Porthidium* specimens from the Field Museum of Natural History (FMNH), the Biodiversity Institute and Natural History Museum of the University of Kansas (KU), the University of Texas at Arlington Amphibian and Reptile Diversity Research Center (UTA), the Louisiana State University Museum of Zoology (LSUMZ), and the Colección Nacional de Anfibios y Reptiles de la Universidad Nacional Autónoma de México (CNAR). Initially, all available specimens were reviewed; however, newborns and specimens with clear damage and misidentified specimens were excluded from the analyses (Table 1).

**Table 1.** Summary of specimens by species and museum collections.^a^

Species	CNAR	FMNH	KU	LSU	UTA
*P. arcosae*	0	0	0	0	1
*P. dunni*	16	1	6	2	8
*P. hespere*	4	0	0	0	0
*P. lansbergii*	0	254	15	0	6
*P. nasutum*	3	25	29	0	25
*P. ophryomegas*	0	1	22	8	16
*P. porrasi*	0	0	0	1	1
*P. volcanicum*	0	0	0	0	1
*P. yucatanicum*	1	29	8	0	1

a Colección Nacional de Anfibios y Reptiles de la Universidad Nacional Autónoma de México (CNAR); Field Museum of Natural History (FMNH); Kansas University Natural History Museum (KU); Museum of Natural Science of the Louisiana State University (LSUMZ); Amphibian and Reptile Diversity Research Center at the University of Texas at Arlington (UTA).

**Table 2.** Description of landmarks of dorsal head *Porthidium* specimens.^a^

Number	Description	Type	LM type
1	Anterior point between the two apical scales	I	LM
2, 6	External margin of apical scales	I	LM
3, 7	Anterior corner of supraocular scale	I	LM
4, 8	Posterior corner of supraocular scale	I	LM
5, 9	External margins of the end of the head	II	sLM
10–12	Outline of the (1)–(2)	III	sLM
13–17	Outline of the (2)–(3)	III	sLM
18–22	Outline of the (3)–(4)	III	sLM
23–32	Outline of the (4)–(5)	III	sLM
33–35	Outline of the (1)–(6)	III	sLM
36–40	Outline of the (6)–(7)	III	sLM
41–45	Outline of the (7)–(8)	III	sLM
46–55	Outline of the (8)–(9)	III	sLM

a ‘Type’ refers to the anatomical classification of landmarks following Bookstein’s criteria: type I landmarks are discrete homologous points (e.g. scale intersections), type II landmarks are points located at maxima of curvature or other defined geometric loci, and type III landmarks are points defined by relative position along a curve between two type I or II landmarks. LM type = landmark time; LM = landmark; sLM = semi-landmark.

**Table 3.** Overview of data files and their content.

File name	Format	Description
Porthidium_morphometrics	CSV	Linear morphometric measurements and specimen metadata
Porthidium_pholidosis	CSV	Meristic scale counts
Porthidium_landmarks	TPS	Raw 2D landmark coordinates for head shape analysis
Porthidium_abbreviation	TXT	Guide to column names and abbreviations

### Morphological traits

**Figure 1.**
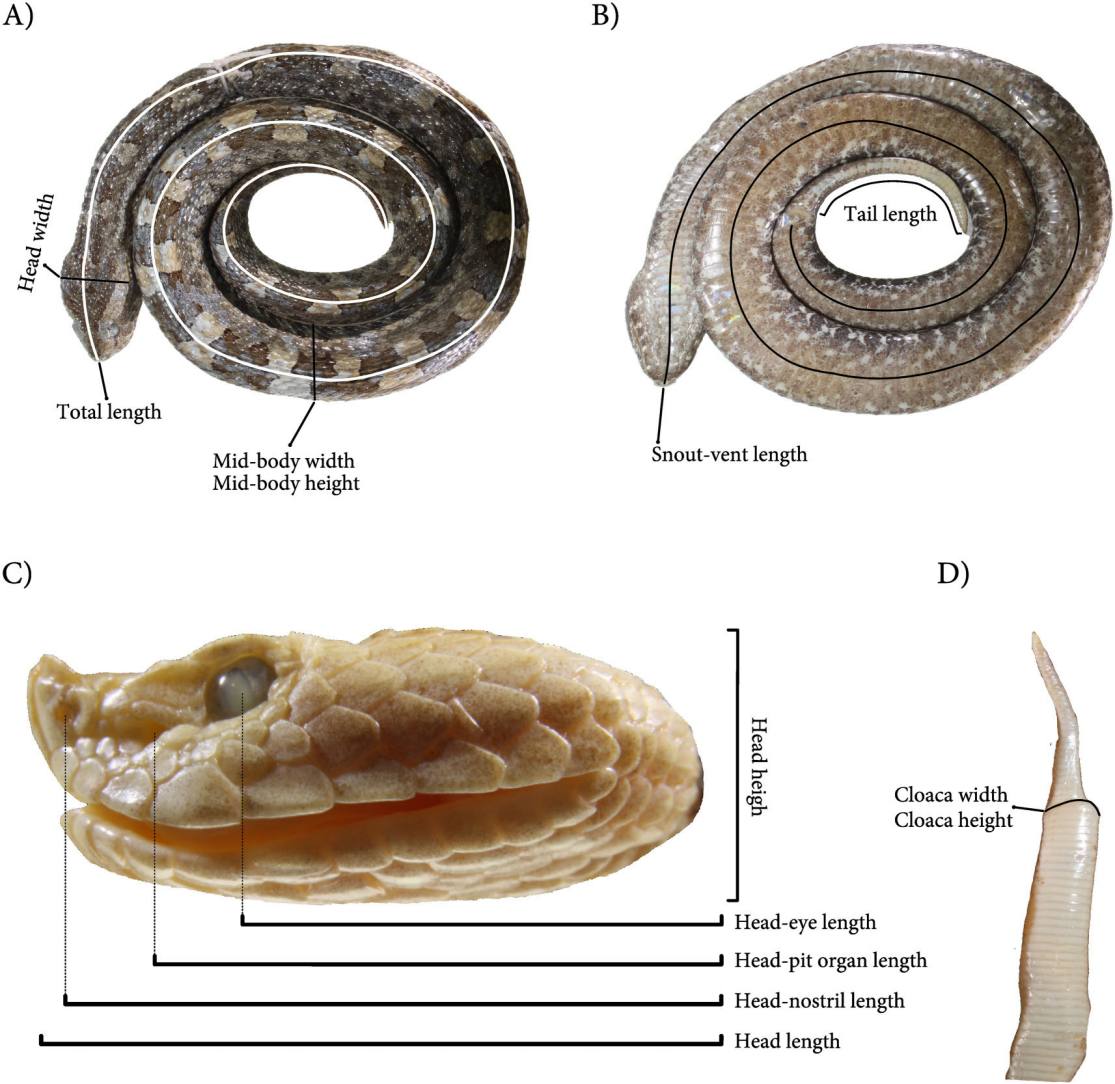
Morphometric traits measured in each *Porthidium* specimen, based on standardized anatomical views. (A) Dorsal view of the whole body showing total length, head width, and mid-body width and height; (B) ventral view highlighting snout–vent length and tail length; (C) lateral view of the head illustrating head height, head length, head to eye length, head to pit organ length, and head to nostril length; and (D) dorsal view of the posterior body showing cloaca width and height.

We collected morphological data following established protocols for viperids [13, 14] to ensure reproducibility and comparability with future studies. Thirteen linear measurements were taken for each specimen using a digital caliper (MarCal 16 EWR; measuring range: 0–150 mm; resolution: 0.01 mm). To minimize measurement error, all recordings were performed by a single experienced observer (P.R.C.), and each measurement was taken twice; when the two measurements differed, the mean was calculated and reported in the database. The measured variables capture key aspects of organismal size and shape: overall size (snout–vent length, total length, tail length), body robustness (mid-body width, mid-body height, cloaca width, cloaca height), head dimensions (head length, head width, head height), and positions of sensory structures (eye-nostril distance, pit-eye distance) (Figure 1). All measurements are reported in millimetres. We acknowledge that soft-tissue metrics are susceptible to known biases from preservation (e.g. tissue contraction, dorsoventral collapse) [15, 16]. While this could alter absolute values, specimens exhibiting clear signs of such distortion involving those metrics were conservatively coded as NA. For the remaining data, we assume that any residual, non-obvious preservation-induced bias is both minor and applied uniformly across specimens. Hence, the relative differences and morphological patterns reported here are expected to be informative and robust [17, 18].

**Figure 2.**
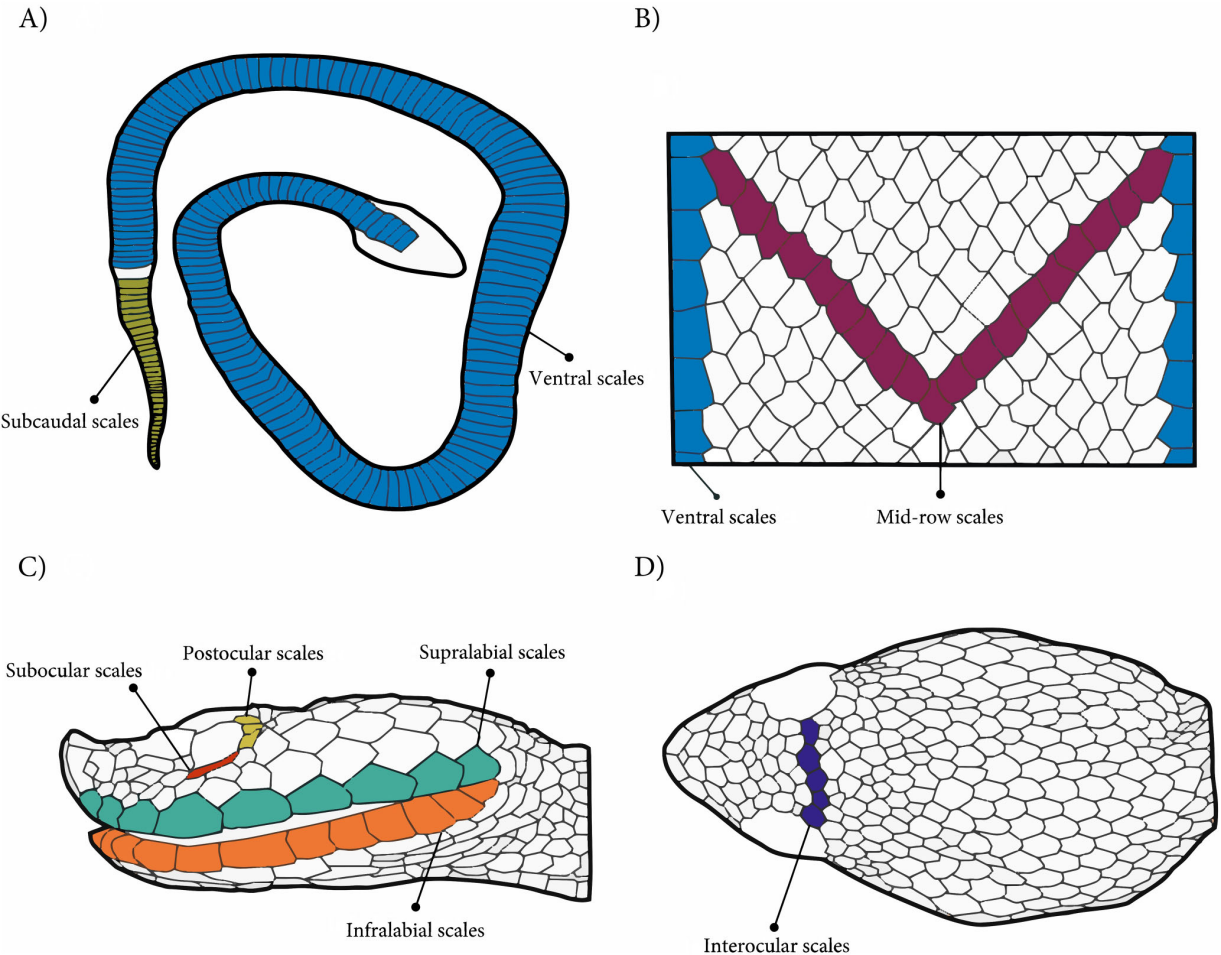
Body and head scales assessed in *Porthidium*. (A) Dorsal view of the entire body, showing the arrangement of ventral and caudal scales. (B) Enlarged dorsal view of the body, highlighting the mid-row dorsal scales and lateral dorsal scales. (C) Lateral view of the head, indicating subocular, postocular, supralabial, and infralabial scales. (D) Dorsal view of the head, showing the interocular scales. Illustration by Carlos Patron-Rivero.

### Scale traits (Pholidosis)

Meristic traits were examined under a stereoscopic microscope (Leica MZ6) and counted according to the standardized methodology [19]. We focused on scale series that are diagnostic in snake taxonomy and are known to exhibit low within-individual variation. The recorded traits encompassed the (1) body scales: ventral scales, subcaudal scales, and the number of dorsal scale rows at mid-body, and (2) cephalic scales: supralabials, infralabials, interoculars, suboculars, and postoculars, which are relevant for prey handling and protection (Figure 2).

**Figure 3.**
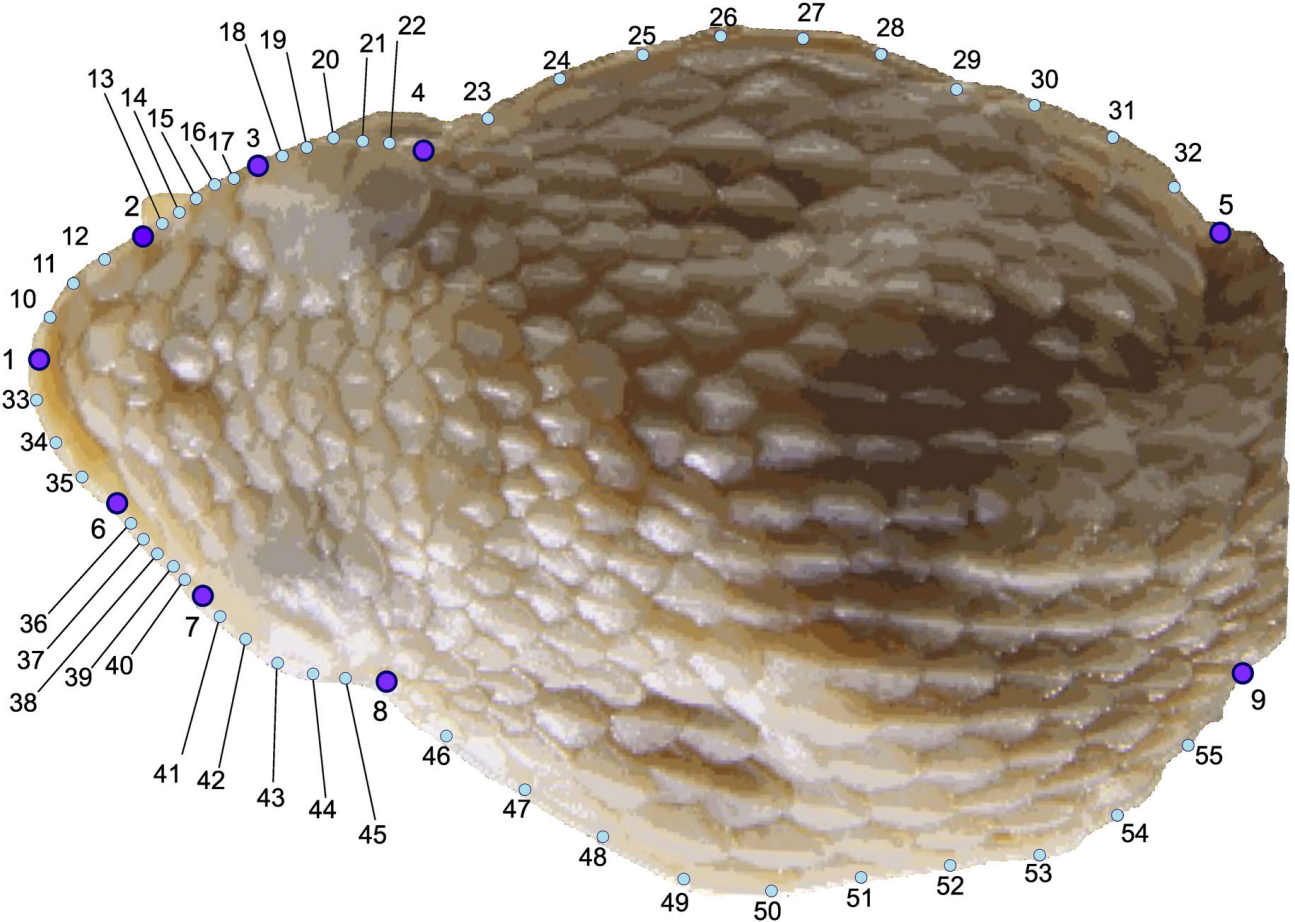
Dorsal view of the head of *Porthidium*, showing the arrangement of landmarks used in geometric morphometric analyses. 1-8 points represent landmarks, while other points represent semi-landmarks derived from curves, placed at equidistant intervals along anatomical edges. Illustration by Carlos Patron-Rivero.

### Shape trait

Each specimen was photographed with a Canon 550D with 4.5-megapixel resolution mounted on a special platform in a dorsal view of the head. A scale bar of 1–20 mm was included in every image. We defined and digitized 55 two-dimensional landmarks and semi-landmarks. The landmark configuration comprised nine fixed anatomical landmarks (types I and II) and 46 semi-landmarks using tpsDig 2.32 (Figure 3; 20). The description of anatomical landmark and semi-landmark organization is in Table 2.

### Maturity

Due to the lack of published data on size at sexual maturity for most *Porthidium* species, we implemented a standardized statistical method to categorize specimens into size classes (juvenile and adult). This was done to provide future users of this database with an objective and reproducible criterion for filtering analyses by size. We applied the Jenks natural breaks optimization algorithm to the snout–vent length (SVL) data using the getJenksBreaks() function in the R package BAMMtools [21]. We emphasize that these categories are statistically defined and should be interpreted as size classes rather than confirmed biological stages.

### Data availability and description

All data described in this manuscript are available and archived on the Zenodo repository (https://doi.org/10.5281/zenodo.16787193) [22]. A dynamic version is also maintained on GitHub (https://github.com/PatronRiveroC/Porthidium_database) for feedback and potential future updates. The dataset is released under a Creative Commons Attribution 4.0 International (CC-BY) licence, allowing for unrestricted use, distribution, and reproduction in any medium, provided the original work is properly cited. The data consist of four primary files that provide a comprehensive quantitative description of the morphology for all examined specimens (Table 3). The *Porthidium_morphology.csv*; this file contains the 13 linear measurements (in millimetres) and associated metadata for each specimen. Key metadata includes museum catalogue number, species identification, geographic locality data (longitude and latitude), and the developmental stage (adult/juvenile) as determined by our criteria. The *Porthidium_pholidosis.csv*; this file contains all meristic scale counts. It includes the number of ventral scales, subcaudal scales, dorsal scale rows at three body positions, and detailed counts of cephalic scales (supralabials, infralabials, interoculars, suboculars, and postoculars). The *Porthidium_landmarks.TPS*; this file contains the raw landmark coordinates for the geometric morphometric analysis of head shape. The file follows the standard TPS format and includes the landmark configurations for all imaged specimens, along with specimen identifiers and scale factors embedded in the file headers. The *Porthidium_abbreviation.txt*; this plain text file serves as a data dictionary. It provides detailed descriptions and definitions for every column header used in the .csv files (Morphometry and Pholidosis).

These data are presented with each row representing a single specimen and each column representing a single variable. This structure facilitates easy ingestion and analysis in various statistical software environments (e.g. R, Python, and PAST). The landmark data (.TPS file) can be read by standard geometric morphometrics software packages such as tpsRelw, MorphoJ, and the R package ‘geomorph’ [23].

## Conclusion

The morphological database for *Porthidium* presented here provides a comprehensive, standardized, and openly accessible resource that directly addresses the pervasive morphological data shortfalls in Neotropical herpetology. By integrating traditional morphometrics, detailed pholidosis, and geometric morphometric data from a wide taxonomic and geographic sample, this dataset offers a quantitative foundation for investigating a multitude of evolutionary and ecological questions in this genus of pitvipers.

The potential applications and reuse of this database are extensive, including but not limited to:

1) *Taxonomic revision and species delimitation*: The high-dimensional phenotypic data provide a robust framework for re-evaluating species boundaries, identifying potential cryptic diversity, and testing the validity of current taxonomic hypotheses within *Porthidium* and related genera.
2) *Phylogenetic comparative methods*: This dataset can be directly integrated with molecular phylogenies to test hypotheses of correlated evolution, assess patterns of morphological disparity, and reconstruct the evolutionary history of key traits, such as head shape and body size.
3) *Ecomorphology and functional morphology*: Researchers can leverage these data to investigate links between morphology and ecology, biomechanical performance of the feeding apparatus, and adaptations to different microhabitats or prey types.
4) *Ontogenetic allometry and developmental studies*: The inclusion of specimens across a range of sizes allows for detailed analyses of allometric trajectories, shedding light on how shape and size change through development and how these patterns vary among species.
5) *Macroecology and biogeography*: Coupled with georeferenced locality data, this morphological resource enables analyses of geographic variation, tests of ecogeographical rules (e.g. Bergmann’s rule), and the exploration of morphological diversity across environmental gradients.

Finally, by depositing the dataset in a repository, we ensure its long-term preservation, stability for reproducible science, and maximum utility for the scientific community. We encourage researchers to utilize this resource, and to contribute to its expansion with additional data, such as sex-specific measurements to explore known patterns of sexual dimorphism [24–26], fostering a collaborative approach to understanding the diversity of Neotropical snakes. Ultimately, this database is not an endpoint but a starting point, designed to future research and serve as a foundational pillar for integrative studies in snake evolution and ecology.
